# Fast phase randomization via two-folds

**DOI:** 10.1098/rspa.2015.0782

**Published:** 2016-02

**Authors:** D. J. W. Simpson, M. R. Jeffrey

**Affiliations:** 1 Institute of Fundamental Sciences, Massey University, Palmerston North, New Zealand; 2 Department of Engineering Mathematics, University of Bristol, Bristol, UK

**Keywords:** piecewise-smooth, desynchronization, Filippov system, sliding motion

## Abstract

A two-fold is a singular point on the discontinuity surface of a piecewise-smooth vector field, at which the vector field is tangent to the discontinuity surface on both sides. If an orbit passes through an invisible two-fold (also known as a Teixeira singularity) before settling to regular periodic motion, then the phase of that motion cannot be determined from initial conditions, and, in the presence of small noise, the asymptotic phase of a large number of sample solutions is highly random. In this paper, we show how the probability distribution of the asymptotic phase depends on the global nonlinear dynamics. We also show how the phase of a smooth oscillator can be randomized by applying a simple discontinuous control law that generates an invisible two-fold. We propose that such a control law can be used to desynchronize a collection of oscillators, and that this manner of phase randomization is fast compared with existing methods (which use fixed points as phase singularities), because there is no slowing of the dynamics near a two-fold.

## 1. Introduction

When an orbit of a system of ordinary differential equations approaches a stable periodic orbit, rather than the location of the orbit in phase space at a given time, a more useful quantity is often the asymptotic (or latent) phase of the orbit. The asymptotic phase distinguishes the long-term behaviour of different orbits, and sets of points with the same asymptotic phase are called *isochrons* [1]. Isochrons cannot intersect, but may accumulate at *phaseless sets* where the asymptotic phase is undefined. Near a point in such a set, termed a *phaseless point*, the asymptotic phase is highly sensitive to perturbations.

In smooth systems, phaseless points are unstable equilibria that can only be approached asymptotically (in backward time). In piecewise-smooth systems, we show that there exist phaseless points that can be intersected in finite time, without any slowing of the dynamics suffered near an equilibrium. The two-fold singularity, an enigma of piecewise-smooth dynamical systems theory, is one such phaseless point.

There are many reasons why it might be desirable to alter the phase of an existing oscillatory motion. One approach to tackling Parkinson’s disease, for instance, is to disturb the synchronized neural activity associated with physical tremors [2,3]. Desynchronization can be achieved with pulses [4], pulse trains [5], time-delayed control [6] or some other well-chosen feedback law [7]. Phase randomization for prototypical neuron models is achieved in [8] by briefly applying a control that transports the orbit to the close proximity of a phaseless point. The orbit subsequently returns to periodic motion but now has a different asymptotic phase. This phase is highly sensitive to the precise point at which the orbit is located when the control is removed, so small stochasticity in the system causes the resulting asymptotic phases of different neurons to be highly randomized.

Two-fold singularities of piecewise-smooth systems were first described by Filippov [9] and have garnered interest as points where both the forward and backward time uniqueness of a flow can break down in an otherwise deterministic flow [10–12]. In a vector field that is discontinuous on some hypersurface—the *discontinuity surface*—a two-fold is a point where the flow has quadratic tangencies (‘folds’) to both sides of the surface. Two-folds occur generically at isolated points in three-dimensional piecewise-smooth vector fields, and on (*n*−2)-dimensional manifolds in *n*-dimensional piecewise-smooth vector fields [12]. They have been identified in models of electronic circuits [13] and contact mechanics [14], and have deep connections to folded nodes of slow–fast systems [15]. The dynamics near a two-fold depends on whether the vector field on either side is curving towards or away from the discontinuity surface, and on the alignments of the two vector fields relative to each other [12].

In this paper, we illustrate the practical implications of a two-fold for phase randomization. As well as suggesting how two-folds might affect real systems, this suggests a use for them as control devices for the desynchronization of oscillators. To this end, we shall consider systems where a two-fold either occurs naturally in a piecewise-smooth system, or is introduced to a smooth system via a discontinuous control. We will simulate orbits in the presence of small noise as they pass close to a two-fold, and focus on how their phases are randomized by the presence of the singularity. The two-fold is not a zero of the vector field, so there is no slowing of the flow during phase randomization achieved in this way.

We shall first demonstrate that the two-fold singularity is a phaseless set, before deriving and simulating its effect on the distribution of the phase as a flow evolves through the singularity. In particular, we show that the distribution of the phase can be manipulated with higher-order terms in the vector field. We also explain how phase randomization can be achieved by using a discontinuous control action to generate a two-fold.

**Figure 1.**
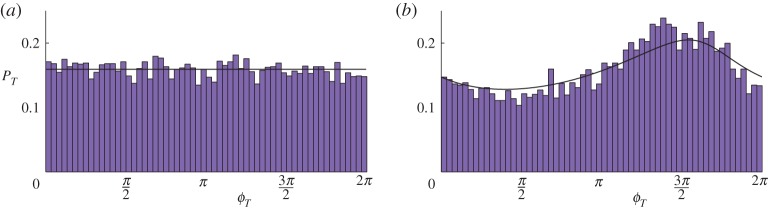
Each panel shows a histogram of the phase *ϕ*_*T*_, of 10^4^ sample solutions from the same initial condition, of the general stochastic system (4.1). The two panels correspond to different choices for the higher-order terms that affect the global dynamics of (4.1). One sample solution corresponding to each panel is shown in figure 7 (see caption of this figure for the parameter values used). The solid curves show our theoretical approximation for the probability density function of *ϕ*_*T*_, see §4b. (Online version in colour.)

Our central mathematical result consists of a simple procedure by which the distribution of the asymptotic phase can be approximated. The results of this approximation are illustrated in figure 1 (to be produced in §4). To produce figure 1, we shall consider the normal form of the two-fold, add higher-order terms such that orbits emanating from the two-fold approach a stable periodic orbit, and add small noise. Figure 1*a* shows a histogram of the phase *ϕ*_*T*_, computed relative to a reference time *T* and limiting to the asymptotic phase as T→∞. This shows an apparently uniform distribution, hence the phase of solutions from the initial point is ‘fully randomized’. Figure 1*b* shows an analogous histogram using different higher-order terms, indicating that nonlinearity in the flow influences the distribution. The black curves show our theoretical approximations for the probability density functions of the phase derived in §4b.

Building on these observations, we propose that a periodic orbit in a smooth system can, via the temporary application of a discontinuous control, be made to take an excursion through a two-fold and then return to periodic motion, but now with a randomized phase. We provide simulations of a simple model in which the effect is to desynchronize a collection of synchronized oscillators. As discussed in §6, two-folds have potential advantages over equilibria as phaseless points in the context of phase randomization. Although we provide a motivating toy example, calculating and optimizing realizable control actions (as in [8]) are beyond the scope of this paper.

In §2, we briefly set out the normal form equations of the invisible two-fold singularity and review a few pertinent details of the local dynamics. Here we include a novel definition of polar coordinates that fit naturally with the local dynamics (and should be of particular interest to dynamicists studying two-folds). We include higher-order terms with the normal form in §3, and extend these definitions before simulating a flow through the two-fold subjected to stochastic perturbations in §4. In §5, we give an example of an application to desynchronize smooth oscillators using a discontinuous control, and provide closing remarks in §6.

## 2. Deterministic dynamics of the normal form

There exist three generic kinds of two-fold singularity, depending on whether both folds are visible (curving away from the discontinuity surface) or invisible (curving towards the discontinuity surface), or one is visible and one is invisible. In each case, there exist local conditions under which the flow traverses the singularity in finite time. In this paper, we focus on the case where both folds are invisible, called an invisible two-fold or Teixeira singularity [10,16], in which the local flow passes through the singularity in finite time. The added interest of the invisible two-fold is that the local flow winds repeatedly around the singularity, giving oscillatory behaviour.

In three dimensions, *X*=(*x*,*y*,*z*), the normal form of the invisible two-fold is the piecewise-smooth system

2.1 $$\dot{X}=\left\{ \begin{array}{ll} F_{L}(X), & x<0,\\ F_{R}(X), & x>0, \end{array}\right.$$

where

**Figure 2.**
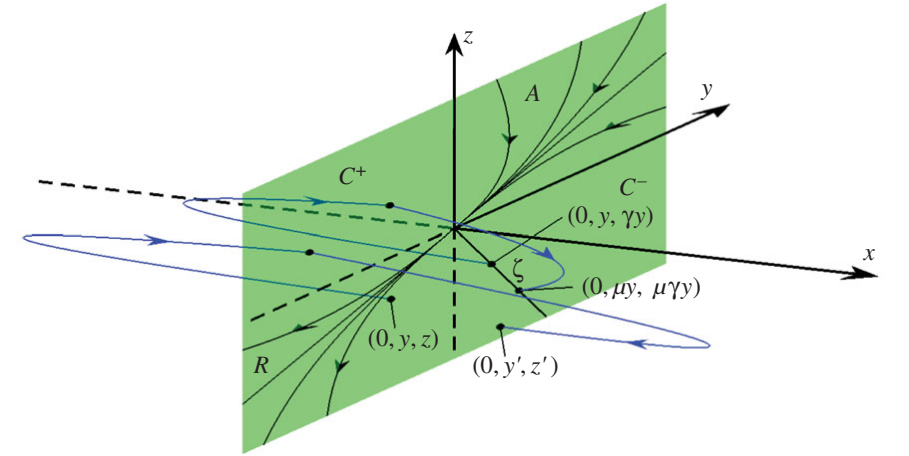
A schematic of the two-fold of (2.1). The discontinuity surface *x*=0 is made up of an attracting sliding region *A* (*y*,*z*>0), a repelling sliding region *R* (*y*,*z*<0), and two crossing sliding regions, *C*^+^ (*y*<0, *z*>0) and *C*^−^ (*y*>0, *z*<0). Parts of two orbits and their intersections with the discontinuity surface are shown. One orbit starts in *R* and has subsequent intersections in *C*^+^ and *C*^−^. A second orbit starts and returns to an invariant line *ζ*∈*C*^−^; see §2a. (Online version in colour.)

2.2 $$F_{L}(X)=(z,V^{-},1),\;F_{R}(X)=(-y,1,V^{+}),$$

and $V^{-},V^{+}\in R$ are parameters [16,17]. The two-fold is located at the origin, *X*=(0,0,0). The discontinuity surface *x*=0 consists of attracting and repelling sliding regions, denoted *A* and *R* (where the flow is confined to the surface and so slides along it), and two crossing regions *C*^±^ (where the flow passes transversally though the surface), as illustrated in figure 2.

The system is solved across the discontinuity by forming the Filippov system for (2.1). Using *e*_*i*_ to denote the *i*th coordinate vector in $R^{3}$, a *Filippov solution* of (2.1) is an absolutely continuous function *φ*(*t*) that satisfies

2.3 $$\left\{ \begin{array}{ll} \frac{d\varphi }{dt}=F_{L}(\varphi (t)), & \mathrm{if}\;e_{1}^{T}\varphi (t)<0,\\ \frac{d\varphi }{dt}=F_{R}(\varphi (t)), & \mathrm{if}\;e_{1}^{T}\varphi (t)>0,\\ \frac{d\varphi }{dt}\in \{(1-q)F_{L}(\varphi (t))+qF_{R}(\varphi (t))\;|\;0\leq q\leq 1\}, & \mathrm{if}\;e_{1}^{T}\varphi (t)=0, \end{array}\right.$$

for almost all values of *t* [9]. We let *φ*(*t*;*X*_0_,*t*_0_) denote a Filippov solution to (2.1) with initial condition *φ*(*t*_0_)=*X*_0_. Each *φ*(*t*;*X*_0_,*t*_0_) is a concatenation of several smooth orbit segments, including segments of evolution outside the discontinuity surface *x*=0, and segments of evolution on *x*=0 referred to as *sliding* motion. Solving the convex combination in (2.3) for $\dot{x}=0$ gives the system that sliding motion obeys on *A* and *R* as

2.4 $$\left[\begin{array}{c} \dot{y}\\ \dot{z} \end{array}\right]\;=\frac{1}{y+z}\;\left[\begin{array}{cc} V^{-} & 1\\ 1 & V^{+} \end{array}\right]\;\left[\begin{array}{c} y\\ z \end{array}\right].$$

Various details of the sliding flow can be derived from (2.4); see [16,17]. Throughout this paper, we assume

2.5 $$V^{-}<0,\;V^{+}<0,\;V^{-}V^{+}>1,$$

in that case a typical orbit of (2.3) and (2.4) will intersect the two-fold in forward time or backward time or both, and give the geometry shown in figure 2. With (2.5), the matrix in (2.4) has distinct negative eigenvalues. Of these, the eigenvalue that is smallest (in absolute value) is $\lambda =\frac{1}{2}(V^{+}+V^{-}+{\left[{\left(V^{+}-V^{-}\right)}^{2}+4\right]}^{1/2})$. As orbits of (2.1) slide into the singularity inside *A*, or slide out of the singularity in *R*, they do so tangent to the weak eigenvector of the matrix (‘weak’ meaning associated with the smallest eigenvalue) and so are tangent to the line *z*=(λ−*V*^−^)*y* on *x*=0. The time to reach or depart the singularity is finite. Specifically, the time is *t*=(*V*^−^−λ−1)*y*/λ from an initial point on the line *z*=(λ−*V*^−^)*y* with *x*=0. Throughout the paper, we use the values

2.6 $$V^{-}=-0.5,\;V^{+}=-2.5,$$

to illustrate the results.

In the remainder of this section, we review crossing solutions to (2.1) in §2a, introduce polar coordinates for describing the flow as it spirals away from the two-folds in §2b, and define the notion of ‘viable’ Filippov solutions in the context of simulation in §2c. In later sections, we apply these concepts to more general piecewise-smooth systems.

### (a) Crossing dynamics and an unstable cone

The left and right half systems of (2.1), $\dot{X}=F_{L}(X)$ and $\dot{X}=F_{R}(X)$, have solutions

2.7 $$\varphi_{L}(t;X_{0},t_{0})=(x_{0}+z_{0}(t-t_{0})+\frac{1}{2}{\left(t-t_{0}\right)}^{2},\;y_{0}+V^{-}(t-t_{0}),\;z_{0}+t-t_{0})$$

and

2.8 $$\varphi_{R}(t;X_{0},t_{0})=(x_{0}-y_{0}(t-t_{0})-\frac{1}{2}{\left(t-t_{0}\right)}^{2},\;y_{0}+t-t_{0},\;z_{0}+V^{+}(t-t_{0})),$$

respectively, where *X*_0_=(*x*_0_,*y*_0_,*z*_0_). In what follows, we use (2.7) and (2.8) to study Filippov solutions *φ*(*t*;0,*y*,*z*,*t*_0_) of (2.1). As indicated in figure 2, orbits wind around regions *A* and *R*, repeatedly crossing *x*=0 in regions *C*^±^. This can be understood with a return map that has been studied in considerable detail [16,18]. Here, we review a few details of this map that are needed for the ensuing analysis.

First consider the region *z*<0 on *x*=0, where the flow *φ*_*L*_ is directed away from the discontinuity surface. If *y*>0, then (0,*y*,*z*)∈*C*^−^, so *φ*_*R*_ is directed towards the surface and hence *φ*(*t*;0,*y*,*z*,*t*_0_) immediately enters *x*<0. If instead *y*<0, then (0,*y*,*z*)∈*R*, so both *φ*_*L*_ and *φ*_*R*_ are directed away from the surface and hence *φ*(*t*;0,*y*,*z*,*t*_0_) is not uniquely determined (it may slide along *x*=0 following (2.4) or enter either *x*<0 or *x*>0 at any instant). For definiteness, when *y*≤0, we consider the Filippov solution *φ*(*t*;0,*y*,*z*,*t*_0_) that immediately enters *x*<0. From (2.7), we find that *φ*(*t*;0,*y*,*z*,*t*_0_) resides in *x*<0 until the later time *t*=*t*_0_−2*z* when it is located at (0,*y*−2*V*^−^*z*,−*z*).

Second, consider *y*<0 and assume *φ*(*t*;0,*y*,*z*,*t*_0_) immediately enters *x*>0. From (2.8), and by applying arguments analogous to those in the previous paragraph, we find that *φ*(*t*;0,*y*,*z*,*t*_0_) resides in *x*>0 until *t*=*t*_0_−2*y* when it is located at (0,−*y*,−2*V*^+^*y*+*z*).

From these observations, we can construct a return map capturing crossing dynamics. This is formulated in the following proposition (refer to [16] for a complete proof).

**Proposition 2.1**

*Suppose V*^−^, *V*^+^<0, *V*^−^*V*^+^>1. *For any* y∈R *and z*<0, *let* (0,*y*′,*z*′) *denote the next intersection of φ*(*t*;0,*y*,*z*,*t*_0_) *with the discontinuity surface at a point with y*′>0 (*assuming φ*(*t*) *immediately enters x*<0 *in the case y*<0). *If z*<2*V*^+^*y*/(4*V*^−^*V*^+^−1), *then z*′<0 *and*

2.9 $$\left[\begin{array}{c} y^{\prime }\\ z^{\prime } \end{array}\right]=\left[\begin{array}{cc} -1 & 2V^{-}\\ -2V^{+} & 4V^{-}V^{+}-1 \end{array}\right]\;[\begin{array}{c} y\\ z \end{array}],$$

*and the evolution time of the orbit from* (*y*,*z*) *to* (*y*′,*z*′) *is*

2.10 $$t-t_{0}=2((2V^{-}-1)z-y),$$

*whereas if z*>2*V*^+^*y*/(4*V*^−^*V*^+^−1), *then z*′>0.

The return map (2.9) is linear, area-preserving, and has a saddle-type fixed point at (*y*,*z*)=(0,0). The unstable multiplier (i.e. the eigenvalue of the matrix in (2.9) with modulus greater than 1) is

2.11 $$\mu =2V^{-}V^{+}\left(1+\sqrt{1-\frac{1}{V^{-}V^{+}}}\right)-1,$$

and the corresponding eigenvector is (1,*γ*), where

2.12 $$\gamma =V^{+}\left(1+\sqrt{1-\frac{1}{V^{-}V^{+}}}\right).$$

In the full three-dimensional space of (2.1), this eigenvector corresponds to a ray

2.13 ζ={(0,y,γy) | y>0},

that emanates from the two-fold, as shown in figure 2.

By evolving the flow forwards from *ζ* according to (2.7) and (2.8), we obtain a surface *Λ* that is given implicitly by

2.14 $$x=\left\{ \begin{array}{ll} \frac{-1}{V^{-}}\Xi (y,z), & x\leq 0,\\ \frac{1}{V^{+}}\Xi (y,z), & x\geq 0, \end{array}\right.$$

where

2.15 $$\Xi (y,z)=\frac{V^{+}y^{2}-2V^{+}V^{-}yz+V^{-}z^{2}}{2(V^{+}V^{-}-1)}.$$

**Figure 3.**
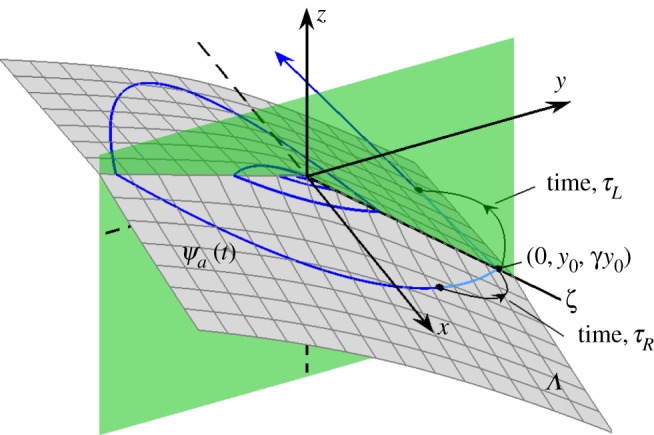
A plot of (2.14) using (2.6). This is the unstable manifold *Λ* (2.14). On *Λ*, one Filippov solution *ψ*_*a*_(*t*) is shown. Formulae for the evolution times *τ*_*L*_>0 and *τ*_*R*_<0 from an arbitrary point (0,*y*_0_,*γy*_0_)∈*ζ* into *x*<0 and *x*>0, respectively, are given by (2.21) and (2.22). (Online version in colour.)

Figure 3 shows a plot of *Λ*. The surface *Λ* is non-differentiable at *x*=0 and consists of part of a hyperbolic paraboloid on each side of *x*=0.

For any initial point on the ray *ζ*, the next intersection of *φ*(*t*;0,*y*,*γy*,*t*_0_) with *C*^−^ is (0,*μy*,*μγy*), shown in figure 2. By substituting *z*=*γy* into (2.10), we find that this iteration of (2.9) corresponds to an evolution time of

2.16 $$t-t_{0}=2((2V^{-}-1)\gamma -1)y.$$

By performing this iteration backwards through repeated crossings, we can construct a Filippov solution that approaches the two-fold in backward time. This Filippov solution emanates from the two-fold and has infinitely many intersections with *ζ* in finite time. Such an orbit is shown in figure 3.

Given any *a*>0, let *ψ*_*a*_(*t*) (defined for *t*≥0) denote the unique Filippov solution that is located at the two-fold at *t*=0, and located on *ζ* at *t*=*a*. That is, *ψ*_*a*_(0)=(0,0,0) and *ψ*_*a*_(*a*)∈*ζ*. By using (2.16) and the classical formula for the sum of a geometric series, we determine the *y*-value of *ψ*_*a*_(*a*) to be

2.17 $$e_{2}^{T}\psi_{a}(a)=\frac{a}{1+\left(1-1/V^{-}\right)/\sqrt{1-1/\left(V^{-}V^{+}\right)}}.$$

After *t*=*a*, the Filippov solution *ψ*_*a*_(*t*) next intersects *ζ* at *t*=*μa*. Consequently, *ψ*_*a*_(*μ*^*n*^*a*)∈*ζ*, for all n∈Z. It follows that each *ψ*_*μ*^*n*^*a*_(*t*) is the same orbit as *ψ*_*a*_(*t*), and so we can characterize all the *ψ*_*a*_(*t*) by restricting our attention to values of *a* in a fundamental domain a*≤*a*<μa*, for some a*>0. Since *Λ* is solely composed of Filippov solutions *ψ*_*a*_(*t*), we can write

2.18 $$\Lambda =\{\psi_{a}(t)\;|\;t\geq 0,1\leq a<\mu \}.$$

The intersection of *Λ* with *C*^−^ is *ζ*. Since *ζ* corresponds to an unstable eigenvector of the crossing map, we refer to *Λ* as an unstable manifold of the two-fold.

### (b) Polar coordinates

Since orbits of (2.1) on *Λ* spiral out from the two-fold (figure 3), it is natural to introduce polar coordinates. The surface *Λ* projects uniquely onto the (*x*,*y*)-plane (and the (*x*,*z*)-plane, but we use the former). The usual polar coordinates (x=rcos⁡(θ), y=rsin⁡(θ)) bear no relation to the behaviour of the system and yield complicated expressions that provide no insight. Instead we derive radial and angular coordinates *r*(*x*,*y*) and *θ*(*x*,*y*) (which we interpret as ‘polar coordinates’) in a way that the restriction of (2.1) to *Λ* takes a particularly simple form.

To obtain a suitable notion of the phase *θ*, we begin by finding the times *τ*_*L*_>0 and *τ*_*R*_<0 that an orbit takes to travel from *ζ* to a given point in *x*<0 and in *x*>0, respectively.

First, choose any *x*<0 and y∈R. We let *y*_0_>0 be such that by evolving (2.1) forwards from (0,*y*_0_,*γy*_0_) we arrive at (*x*,*y*,*z*)∈*Λ*, for some z∈R, without again intersecting *x*=0, and let *τ*_*L*_>0 be the corresponding evolution time. This is illustrated in figure 3. Note that the value of *z* is given from (2.14) by *x*=−*Ξ*(*y*,*z*)/*V*^−^.

We have *φ*_*L*_(*τ*_*L*_;0,*y*_0_,*γy*_0_,0)=(*x*,*y*,*z*), and so, by (2.7),

2.19 $$x=\gamma y_{0}\tau_{L}+\frac{1}{2}\tau_{L}^{2},\;y=y_{0}+V^{-}\tau_{L}\;\mathrm{and}\;z=\gamma y_{0}+\tau_{L}.$$

We solve the first two of these equations simultaneously for *y*_0_ and *τ*_*L*_. Eliminating *y*_0_ yields $(\frac{1}{2}-\gamma V^{-})\tau_{L}^{2}+\gamma y\tau_{L}-x=0$, to which an application of the quadratic formula gives

2.20 $$\tau_{L}=\frac{y-\sqrt{y^{2}-2(2\gamma V^{-}-1)x/\gamma^{2}}}{(2\gamma V^{-}-1)/\gamma }.$$

Upon substituting (2.12) into (2.20), substantial simplification is possible and we obtain

2.21 $$\tau_{L}=\frac{y-\sqrt{y^{2}-(2V^{-}/V^{+})x}}{V^{-}\left(1+\sqrt{1-1/\left(V^{-}V^{+}\right)}\right)}.$$

Moreover, *y*_0_ is given by *y*_0_=*y*−*V*^−^*τ*_*L*_ with (2.21).

Second, choose any *x*>0 and y∈R. We let *y*_0_>0 be such that by evolving (2.1) backwards from (0,*y*_0_,*γy*_0_) we arrive at (*x*,*y*,*z*)∈*Λ*, for some z∈R, without first re-intersecting *x*=0, and let *τ*_*R*_<0 be the corresponding evolution time. Here *z* is specified by *x*=*Ξ*(*y*,*z*)/*V*^+^, and, in the same manner as above, we obtain

2.22 $$\tau_{R}=y-\sqrt{y^{2}+2x},$$

and *y*_0_=*y*−*τ*_*R*_.

Using these expressions, we now build a useful set of polar coordinates for the projection of *Λ* onto the (*x*,*y*)-plane. We let the positive *y*-axis correspond to *θ*=0 and *r*=*y*, i.e.

2.23 r(0,y)=y,θ(0,y)=0,for all y>0.

The positive *y*-axis corresponds to points on *ζ*. Naturally, we want to define *θ* such that a change of 2*π* corresponds to one complete revolution from *ζ* back to itself. By (2.16), forward evolution from (0,*y*,*γy*) returns to *ζ* in a time proportional to *y*. This suggests that we want $\dot{\theta }$ to be inversely proportional to *y* (or the distance from the two-fold, *r*). Moreover, the orbit returns to *ζ* at the point (0,*μy*,*μγy*). That is, the value of *r* increases from *y* to *μy* and so changes by an amount proportional to *y*, suggesting $\dot{r\;}\;$ should be constant. In summary, we would like to define *r* and *θ* so that they satisfy (2.23) and

2.24 $$\dot{r\;}\;=\alpha ,\;\dot{\theta }=\frac{\beta }{r},$$

for some constants *α*,*β*>0. The following choice of *r* and *θ* achieves this.

**Proposition 2.2**

*Suppose V*^−^, *V*^+^<0 *and V*^−^*V*^+^>1. *Let*

2.25 $$r(x,y)=\left\{ \begin{array}{ll} \alpha y-(\alpha -1)\sqrt{y^{2}-\frac{2V^{-}}{V^{+}}x}, & x<0,\\ \alpha y-(\alpha -1)\sqrt{y^{2}+2x}, & x>0, \end{array}\right.$$

*and*

2.26 $$\theta (x,y)=\left\{ \begin{array}{ll} \frac{\beta }{\alpha }\ln\left(1+\frac{\alpha }{y/\tau_{L}(x,y)-V^{-}}\right), & x<0,\\ \frac{\beta }{\alpha }\ln\left(1+\frac{\alpha }{y/\tau_{R}(x,y)-1}\right)+2\pi , & x>0, \end{array}\right.$$

*where τ*_*L*_ *and τ*_*R*_ *are given by* (**2.21**) *and* (*2.22*), *and*

2.27 $$\alpha =\frac{1}{1+\left(1-1/V^{-}\right)/\sqrt{1-1/\left(V^{-}V^{+}\right)}}\;\mathrm{and}\;\beta =\frac{2\pi \alpha }{\ln(\mu )},$$

*where μ is given by* (*2.11*). *Then* (*2.25*) *and* (*2.26*) *define a continuous bijection from* $(x,y)\in R^{2}$ *to* (r,θ)∈(0,∞)×[0,2π)∪(0,0) *that satisfies* (*2.23*). *Moreover, under this coordinate change the restriction of* (*2.1*) *to Λ is given by* (*2.24*) *with* (*2.27*).

**Figure 4.**
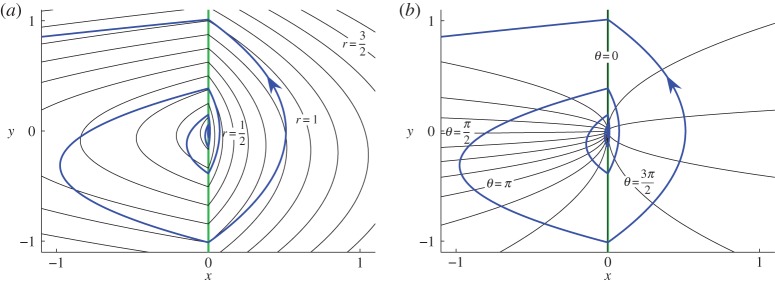
Contours *r*(*x*,*y*)=*constant* (panel *a*) and *θ*(*x*,*y*)=*constant* (panel *b*), where *r* and *θ* are given by (2.25) and (2.26). Contours of *θ*(*x*,*y*) are parabolas of the form *x*=*Ky*^2^, for different values of *K*. Contours of *r*(*x*,*y*) are parabolas of the form *x*=*c*_1_(*y*−*K*)^2^+*c*_2_*K*^2^, for different values of *K*, where *c*_1_ and *c*_2_ are constants that depend on the values of *V*^−^ and *V*^+^. In each plot, one orbit of (2.1) is shown spiralling away from the origin. These illustrations use (2.6) for the values of *V*^−^ and *V*^+^. (Online version in colour.)

A constructive proof of proposition 2.2 using (2.7) and (2.8) is given in appendix A. Figure 4 illustrates contours of *r*(*x*,*y*) and *θ*(*x*,*y*) as defined in (2.25) and (2.26).

Given (2.26), the negative *y*-axis corresponds to θ=2πln⁡((1−2α)μ)/ln⁡(μ). Also by (2.27), we have $0<\alpha <\frac{1}{2}$, with *α*→0 as *V*^−^*V*^+^→1 and $\alpha \to \frac{1}{2}$ as $V^{-}V^{+}\to \infty$ (assuming *V*^−^/*V*^+^ is fixed as the limit is taken). Graphically, we have found also that 0<*β*<*π*.

The right-hand side of (2.24) is defined everywhere except at *r*=0 (the two-fold) and can be solved explicitly. Taking *r*→0 as *t*→*t*_0_ as an initial condition, for all *t*>*t*_0_ the solution to (2.24) is given by

2.28 $$r(t)=\alpha (t-t_{0}),\;\theta (t)=\left(\frac{\beta }{\alpha }\ln(t-t_{0})+C\right)\mathrm{mod}\;2\pi ,$$

for some *C*∈[0,2*π*). Therefore, the Filippov solution *ψ*_*a*_(*t*), defined in §2a, may be written in polar coordinates as

2.29 $$\psi_{a}(t)=\left(r(t),\theta (t)\right)=\left(\alpha t,\;\frac{\beta }{\alpha }\ln\left(\frac{t}{a}\right)\mathrm{mod}\;2\pi \right).$$

### (c) Viable Filippov solutions

The following result shows that orbits emanating from the two-fold either dwell in the repelling sliding region *R* for some time, or evolve on *Λ* for all later times.

**Proposition 2.3**

*Let φ*(*t*)=*φ*(*t*;0,0,0,0) *be a Filippov solution of* (*2.1*) *located at the two-fold at t*=0. *Then either*: (*i*) *φ*(*t*)∈*Λ* *for all t*>0 *or* (*ii*) *φ*(*t*)∈*R* *for some t*>0 *and φ*(*t*)∉*Λ for all t*>0.

**Proof.**

First, suppose *φ*(*t*)∈*R* for some *t*>0. Since Filippov solutions to (2.1) can only enter the repelling region *R* by passing through the two-fold (illustrated in figure 2), then either *φ*(*t*)∈*R* for all *t*>0, in which case clearly *φ*(*t*)∉*Λ* for all *t*>0, or *φ*(*t*)∈*R* on time interval (0,*b*], and at *t*=*b*, the Filippov solution *φ*(*t*) is ejected into either *x*<0 or *x*>0. Suppose without loss of generality that *φ*(*t*) enters *x*<0. Then, subsequent crossing motion (for all *t*≥0, it can be shown [12]) is described by the return map (2.9) starting from *φ*(*b*). Since *φ*(*b*)∉*ζ*, iterations under (2.9) do not lie on *ζ*, hence *φ*(*t*)∉*Λ* for all *t*>0.

Second, suppose *φ*(*t*)∉*R* for all *t*>0. By solving backwards in time, the only way that solutions can reach the two-fold without intersecting *R* is through intersections with *ζ* (illustrated in figure 3). This is because orbits cannot reach the two-fold by evolving backwards in time on the attracting sliding region. Nor can solutions reach the two-fold by evolving purely in either the left half-space (*x*≤0) or the right half-space (*x*≥0) because such evolution follows (2.7) and (2.8). The only remaining possibility is to reach the two-fold via crossing motion. In view of the saddle-type nature of (*y*,*z*)=(0,0) for (2.9), this can only be achieved along the unstable eigenvector. Therefore *φ*(*t*)∈*Λ* for all *t*>0. ▪

Now, we introduce the notion of a ‘viable’ Filippov solution to represent an orbit that is robust for the purpose of forward time numerical simulation. Any orbit that evolves from a point in *R*, simulated using discretization or by modelling the switch using hysteresis, time-delay or noise, is likely to be immediately ejected into either *x*<0 or *x*>0. In view of proposition 2.3, any simulated orbit that arrives at the two-fold is likely to subsequently evolve on *Λ* (or at least very near *Λ*), as observed in [16]. Indeed, perturbations of a planar two-fold by hysteresis, time-delay and noise studied in [19] confirm that perturbed systems follow Filippov solutions provided they do not lie on a repelling sliding region.

**Definition 2.1**

A Filippov solution is said to *viable* if it does not intersect (or travel along) a repelling sliding region in forward time.

For the system (2.1), *φ*(*t*;*X*_0_,*t*_0_) is viable if and only if, for all *t*>*t*_0_, *φ*(*t*;*X*_0_,*t*_0_)∉*R*. The idea is that viable Filippov solutions are the most relevant in real systems or in simulations. The last result of this section follows immediately from proposition 2.3 and provides us with a complete characterization of viable Filippov solutions to (2.1) that pass through the two-fold.

**Corollary 2.1**

*Let φ*(*t*)=*φ*(*t*;0,0,0,0) *be a Filippov solution of* (*2.1*) *that is located at the two-fold at t*=0. *Then φ*(*t*) *is viable if and only if φ*(*t*)=*ψ*_*a*_(*t*), *for some a.*

## 3. Deterministic global dynamics

The vector field near a generic invisible two-fold in a three-dimensional system can be transformed to

3.1 $$\dot{X\;}\;=\left\{ \begin{array}{ll} F_{L}(X)+G_{L}(X), & x<0,\\ F_{R}(X)+G_{R}(X), & x>0, \end{array}\right.$$

where *F*_*L*_ and *F*_*R*_ are the constituents of the normal form (2.2), and

3.2 $$G_{L,R}(X)=(O(x,|y,z{|}^{2}),O(|X|),O(|X|))$$

represent higher-order terms.

As described in [16], there exists a surface $\tilde{\Lambda }$ (that coincides with *Λ* in the limit |*X*|→0) on which viable Filippov solutions to (3.1) evolve. This surface intersects *x*=0 with *y*>0 on a curve $\tilde{\zeta }$ that matches *ζ* to first order. We let ${\tilde{\psi }}_{a}(t)$ denote a viable Filippov solution to (3.1) with ${\tilde{\psi }}_{a}(0)=0$ and ${\tilde{\psi }}_{a}(a)\in \tilde{\zeta }$.

Next, in §3a, we define the asymptotic phase for orbits of (3.1), study the associated isochrons in §3b, and find times of return to the discontinuity surface in §3c.

### (a) The asymptotic phase of an orbit

Let us suppose that (3.1) has a stable periodic orbit *Γ* of period *τ* such that orbits emanating from the two-fold on $\tilde{\Lambda }$ approach *Γ*. Examples are given below and also in [16]. In the examples considered below, *Γ* has exactly two intersections with the discontinuity surface, one with *y*>0 and one with *y*<0. We use the intersection point with *y*>0 as a reference point with zero phase.

Let $\tilde{\varphi }(t;X_{0},t_{0})$ be a Filippov solution to (3.1) that limits to *Γ* as t→∞. The *phase* of $\tilde{\varphi }$, relative to a reference time *T* that is assumed to be sufficiently large that the orbit lies close to *Γ*, is defined as

3.3 $$\phi_{T}=\frac{2\pi (T-s_{T})}{\tau }\;\mathrm{mod}\;2\pi \;\mathrm{and}\;s_{T}=\max_{t\leq T}[x(t)=0,\;y(t)>0].$$

Note that *s*_*T*_≤*T* is the previous time at which the orbit lies on *x*=0 with *y*>0. The ‘*mod* 2*π*’ ensures *ϕ*_*T*_=[0,2*π*), but is almost redundant, because the orbit is close to *Γ* and so *T*−*s*_*T*_ is unlikely to be greater than *τ*. Assuming the forward orbit of *X*_0_ is unique and converges to *Γ*, the *asymptotic phase* of *X*_0_ is defined as

3.4 $$\phi =\lim_{T\to \infty }\phi_{T}.$$

### (b) Isochrons

**Figure 5.**
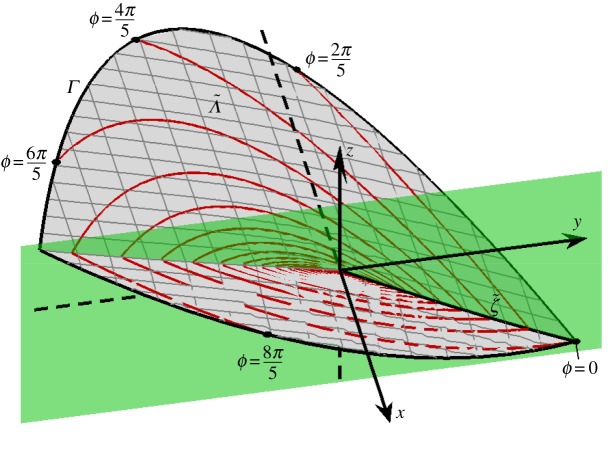
The intersection of five isochrons with the surface $\tilde{\Lambda }$ for the system (3.1) with (2.6) and (3.5). The phases of the isochrons are indicated. (Online version in colour.)

An *isochron* is a set of points with the same asymptotic phase [1]. Isochrons were introduced by Winfree in [20] and shown to be (*n*−1)-dimensional *C*^*k*^ manifolds for *n*-dimensional *C*^*k*^ vector fields by Guckenheimer in [21]. Isochrons of the three-dimensional piecewise-smooth system (3.1) are therefore two-dimensional piecewise-smooth manifolds. Since we are only concerned with orbits that spiral out from the two-fold, it suffices to consider the intersection of the isochrons of (3.1) with $\tilde{\Lambda }$. Figure 5 shows the intersection of five isochrons with $\tilde{\Lambda }$, produced using

3.5 $$G_{L}(X)=G_{R}(X)=-X.$$

The surface $\tilde{\Lambda }$ was computed by fitting a mesh to 1000 numerically computed forward orbits. The stable periodic orbit *Γ* forms the boundary of this surface. The isochrons were computed by interpolating between computed points on the forward orbits and correspond to *ϕ*=2*πk*/5 for *k*=0,…,4. This method is described by Winfree in [1]. More sophisticated methods for computing isochrons are discussed in [22,23].

The isochrons accumulate at the two-fold where the asymptotic phase is undefined. The two-fold is a phaseless point and the basin of attraction of the two-fold, which is a three-dimensional region, is a phaseless set.

### (c) Times of return to the discontinuity surface

**Figure 6.**
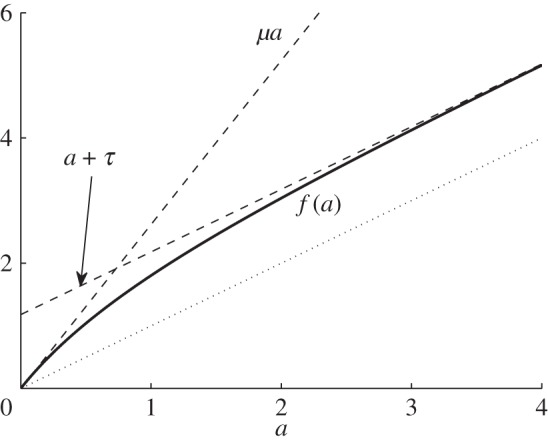
The return time function *f* for the system (3.1) with (2.6) and (3.5).

Here, we define a return time function *f* for the curve $\tilde{\zeta }$. First note that ${\tilde{\psi }}_{a}(a)\in \tilde{\zeta }$ by definition. Then, for any *a*>0, let *t*=*f*(*a*)>*a* be the next time at which ${\tilde{\psi }}_{a}(t)\in \tilde{\zeta }$. Figure 6 shows a plot of *f* using (2.6) and (3.5) and was computed by numerical simulation.

For small *a*, *f*(*a*)≈*μa*, because near the two-fold the higher-order terms *G*_*L*_ and *G*_*R*_ have little effect and the return time is *μa* for the normal form (2.1), as stated in §2a. For large *a*, ${\tilde{\psi }}_{a}(t)$ is located near *Γ* and so *f*(*a*)≈*a*+*τ*.

In §4, we use *f* to extrapolate the probability density function for *ϕ*_*T*_ from points near the two-fold to points near *Γ*.

## 4. Stochastic dynamics and phase randomization

Here, we study a stochastic perturbation of (3.1) defined by the three-dimensional stochastic differential equation

4.1 $$dX(t)=\left\{\begin{array}{cc} F_{L}(X(t))+G_{L}(X(t)), & x(t)<0\\ F_{R}(X(t))+G_{R}(X(t)), & x(t)>0 \end{array}\right\}dt+\varepsilon D\;dW(t),$$

where *W*(*t*) is a standard three-dimensional Brownian motion, *D* is a 3×3 matrix of constants, and 0≤*ε*≪1 represents the noise amplitude. Given a sample solution to (4.1), ${\tilde{\varphi }}_{\varepsilon }(t;X_{0},0)$, and a time *T*, we define *s*_*T*_ and *ϕ*_*T*_ by (3.4) in the same manner as for the deterministic system (3.1).

Here we show that the asymptotic phase is highly randomized for sample solutions to (4.1) that pass close to the two-fold before approaching a stable periodic orbit. We provide numerical evidence for this in §4a, then derive an approximation to the phase distribution in §4b. To illustrate the results, we use (2.6) and two choices for *G*_*L*_ and *G*_*R*_, namely (3.5) and

4.2 $$G_{L}(X)=G_{R}(X)=(-x^{3},-y^{3},0),$$

because they provide substantially different phase distributions. With different values of *V*^−^ and *V*^+^ and different choices for *G*_*L*_ and *G*_*R*_, we have observed similar results.

### (a) Sample solutions and phase randomization

**Figure 7.**
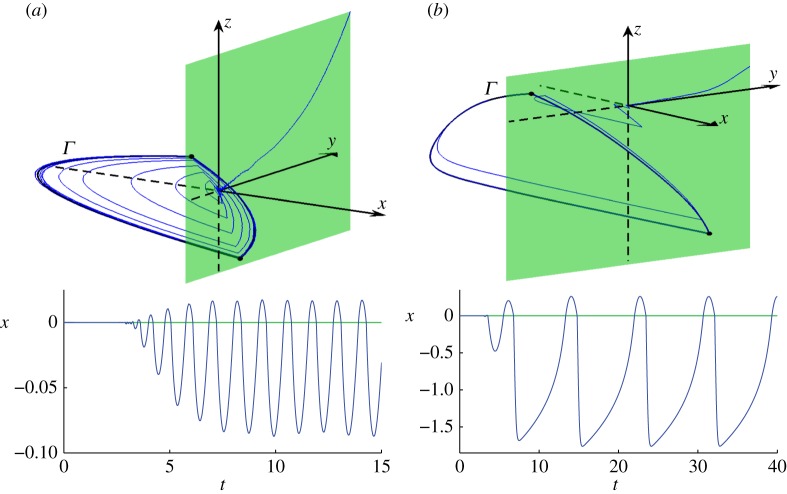
(*a*) A typical sample solution, both in phase space and as a time series, for (4.1) with (2.6) and (3.5) using *D*=*I* and *ε*=0.001. (*b*) A typical sample solution using instead (4.2). Both solutions start at *X*=(0,1,1), pass near the two-fold *X*=(0,0,0), then approach a stable periodic orbit *Γ*. The solutions were computed using the Euler–Maruyama method with a step-size of Δ*t*=10^−5^. (Online version in colour.)

A sample solution to (4.1) using (2.6) and (3.5) is shown in figure 7*a*. The initial point is *X*=(0,1,1), and we denote this solution as ${\tilde{\varphi }}_{\varepsilon }(t;0,1,1,0)$. The deterministic solution $\tilde{\varphi }(t;0,1,1,0)$ slides into the two-fold in a time *t*_0_=3.0445 (to four decimal places). The perturbed solution ${\tilde{\varphi }}_{\varepsilon }(t)$ initially follows a random path close to $\tilde{\varphi }(t)$ near the discontinuity surface. Then while *t*≈*t*_0_, the perturbed solution ${\tilde{\varphi }}_{\varepsilon }(t)$ is located near the two-fold. It is during this brief period that the order-*ε* noise has an order-1 influence on the location and phase of ${\tilde{\varphi }}_{\varepsilon }(t)$ at later times. Next ${\tilde{\varphi }}_{\varepsilon }(t)$ spirals outwards. The initial portion of this spiralling motion is close to the two-fold, and so the higher-order terms *G*_*L*_ and *G*_*R*_ have little influence. At later times, ${\tilde{\varphi }}_{\varepsilon }(t)$ is located relatively far from the two-fold. Here *G*_*L*_ and *G*_*R*_ are important and ${\tilde{\varphi }}_{\varepsilon }(t)$ approaches the stable periodic orbit of (3.1), *Γ*.

We computed 10^4^ sample solutions analogous to the solution shown in figure 7*a* (i.e. with the same initial point and parameter values). Figure 1*a* shows a histogram of the phase *ϕ*_*T*_ of these solutions. Here we used *T*=15, because transient dynamics appears to have decayed by this time. We note that the phase is roughly uniformly distributed on [0,2*π*).

Figure 7*b* shows a sample solution using (4.2) instead of (3.5). As in figure 7*a*, the solution approaches the two-fold (the deterministic sliding time is *t*_0_=2.9763, to four decimal places), then spirals out towards a stable periodic orbit *Γ*. Figure 1*b* shows a histogram of the phase *ϕ*_*T*_ of 10^4^ sample solutions, using *T*=40. Again, the phase is highly random, but, in this case, the phase distribution is not well approximated by a uniform distribution. A phase of *ϕ*_*T*_≈3*π*/2 appears to be about twice as likely as a phase of *ϕ*_*T*_≈*π*/2. In further simulations (not shown) with different values of *ε* and *T*, we observed similar phase distributions to those shown in figure 1.

In §4a, we combine the polar coordinate description of the local dynamics (given in §2b) with the global return time function *f* (given in §3c) in order to construct approximations to the phase distributions of figure 1.

### (b) The probability distribution of the asymptotic phase

Let $X_{0}\in R^{3}$ be such that the deterministic forward orbit of this point $\tilde{\varphi }(t;X_{0},0)$ is located at the two-fold at some time *t*_0_>0. For sample solutions ${\tilde{\varphi }}_{\varepsilon }(t;X_{0},0)$, we are only interested in the phase *ϕ*_*T*_ at large values of *T* (in order to adequately approximate the asymptotic phase *ϕ*) but our analysis requires considering all *T*>*t*_0_.

For any *T*>*t*_0_, let *p*_*T*_(*s*_*T*_) denote the probability density function for the value of *s*_*T*_ (the previous time of intersection with the discontinuity surface at a point with *y*>0). We begin by explaining that for intermediate values of *T*−*t*_0_, specifically *ε*≪*T*−*t*_0_≪1, it is suitable to assume that *s*_*T*_−*t*_0_ has a reciprocal probability distribution, that is,

4.3 $$p_{T}(s_{T})=\frac{1}{\ln(\left(T-t_{0}\right)/\left(f^{-1}(T)-t_{0}\right))(s_{T}-t_{0})}.$$

Since the noise amplitude *ε* is small, if *T*−*t*_0_≪1 then an arbitrary sample solution ${\tilde{\varphi }}_{\varepsilon }(t)$ will lie near the two-fold with high probability. Thus for the purposes of computing *s*_*T*_, we can ignore the higher-order terms *G*_*L*_ and *G*_*R*_. Also if *ε*≪*T*−*t*_0_, then with high probability an arbitrary sample solution will lie sufficiently far from the two-fold that for the purposes of computing *s*_*T*_ we can ignore the noise. Therefore, here it is suitable to restrict our attention to the normal form (2.1), and we work with this system in polar coordinates (2.24).

Any solution to (2.24) that limits to the two-fold as *t*→*t*_0_ (with *t*>*t*_0_) is given by (2.28) for some *C*∈[0,2*π*). The system (2.24) is independent of *θ*, which implies we should treat *C* as a random variable with a uniform distribution on [0,2*π*).

Given *C*, we can calculate *s*_*T*_ by using (2.28) to solve *θ*(*t*)=0 for *s*_*T*_. We write $(\beta /\alpha )\ln(T-t_{0})=2\pi n+\hat{\theta }$, for some n∈Z and $\hat{\theta }\in [0,2\pi )$. By (2.28), if $0\leq C<2\pi -\hat{\theta ,}$ then

4.4 $$\frac{\beta }{\alpha }\ln(s_{T}-t_{0})+C=2\pi n,$$

and if $2\pi -\hat{\theta }\leq C<2\pi ,$ then

4.5 $$\frac{\beta }{\alpha }\ln(s_{T}-t_{0})+C=2\pi (n+1).$$

By (4.4) and (4.5), the assumption that *C* is uniformly distributed implies that $\ln(s_{T}-t_{0})$ is also uniformly distributed. Therefore, in this approximation, *s*_*T*_−*t*_0_ has a reciprocal probability distribution as given by (4.3).

As sample solutions ${\tilde{\varphi }}_{\varepsilon }(t)$ continue to evolve outwards from the two-fold, because there are no other singular points for solutions to encounter, the noise only has the effect of diffusing intersection times *s*_*T*_ by a small amount. Therefore, in order to approximate *p*_*T*_ for larger values of *T*>*t*_0_, we can again ignore the noise (as long as *T* is not so large that the contribution of the accumulated small diffusive effects is significant). In this approximation, *p*_*T*_ can be expressed in terms of *p*_*f*^−1^(*T*)_ by iterating the density under *f*, specifically

4.6 $$p_{T}(s_{T})=p_{f^{-1}(T)}(f^{-1}(s_{T}))\frac{df^{-1}}{ds_{T}}.$$

Iterating *n* times gives

4.7 $$p_{T}(s_{T})=p_{f^{-n}(T)}(f^{-n}(s_{T}))\frac{df^{-n}}{ds_{T}}.$$

We can therefore approximate *p*_*T*_ for a large value of *T* by using (4.7) with a value of *n* that is sufficiently large that *f*^−*n*^(*T*)−*t*_0_ is small, and so the reciprocal probability density function (4.3) can be used for *p*_*f*^−*n*^(*T*)_. This is the manner by which we obtained the two approximations shown in figure 1, using *n*=10 in both cases. The iterative procedure (4.7) was performed numerically, because analytic expressions for *f* appear to be unavailable.

**Figure 8.**
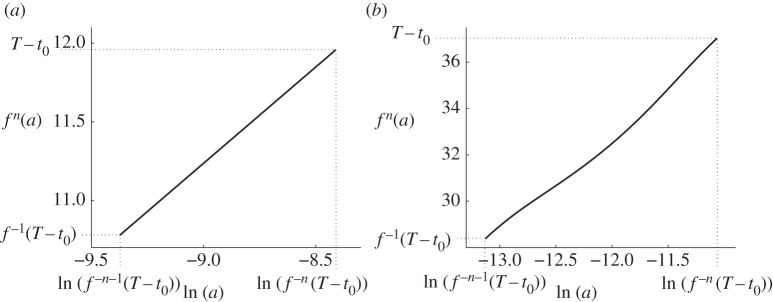
Plots of *f*^*n*^, using *n*=10, where *f* is the return time function defined in §3c. Panel (*a*) corresponds to (3.5), for which *t*_0_≈3.0445, and we used *T*=15. Panel (*b*) corresponds to (4.2), for which *t*_0_≈2.9763, and we used *T*=40.

Figure 8 shows plots of *f*^*n*^ for both (3.5) and (4.2). We note that if *f*^*n*^(*a*) is an affine function of ln⁡(a) then our procedure generates a uniform distribution for *p*_*T*_. Indeed, in figure 8*a*, *f*^*n*^ is indistinguishable from an affine function on the given scale and so, in figure 1*a*, the distribution is approximately uniform. In figure 8*b*, *f*^*n*^ has a notable nonlinearity, and, for this reason, the distribution in figure 1*b* is significantly non-uniform.

## 5. Desynchronizing a collection of smooth oscillators

Now suppose that we wish to break the synchrony of a collection of coordinated oscillators. In [8], this is achieved by applying a control that pushes the state of the oscillators towards an unstable equilibrium, where small random perturbations efficiently randomize the phase of the oscillators. Upon removal of the control action, each oscillator returns to the original regular periodic motion but now the oscillators are desynchronized. Here we propose a ‘fast’ method to achieve this, using a two-fold as the phase singularity rather than an equilibrium; in that case there is no slowing of the dynamics as the oscillators return to periodic motion. The example we give is intended to illustrate this concept in a simple way, with practical applications left to future work.

To illustrate our method, we assume the oscillators are governed by the planar system

5.1 $$(\dot{x\;}\;,\dot{y\;}\;)=F(x,y)=(x-y-x(x^{2}+y^{2}),x+y-y(x^{2}+y^{2})),$$

and apply a discontinuous control that creates a two-fold singularity. In principle, this can be achieved with any system exhibiting a stable periodic orbit, including excitable systems such as the FitzHugh–Nagumo equations [8].

Consider the two-dimensional stochastic differential equation

5.2 $$(dx(t),dy(t))=(F(x(t),y(t))+H(t-t_{1})H(t_{2}-t)c(t))\;dt+\varepsilon \;dW(t),$$

where *W*(*t*) is a standard two-dimensional Brownian motion, and 0≤*ε*<1 is the noise amplitude. The function *H* is the Heaviside function and *t*_1_ and *t*_2_ are the start and end times of the control action *c*(*t*). In order to generate a generic two-fold, *c*(*t*) must be discontinuous and time-dependent. Here we show that the piecewise-linear form

5.3 $$c(t)=\left\{ \begin{array}{ll} (a_{1}t,a_{2}), & x\leq 0,\\ (a_{3}t,a_{4}), & x>0, \end{array}\right.$$

where $a_{1},a_{2},a_{3},a_{4}\in R$ are control parameters, is sufficient for our purposes. An identification of more sophisticated functions providing superior performance is beyond the scope of this paper.

By treating the time *t* as a third dynamic variable, i.e. with $\dot{t\;}\;=1$, the system is three-dimensional and is piecewise-smooth when *t*_1_<*t*<*t*_2_ with the discontinuity surface *x*=0. The fold lines on *x*=0 (where $\dot{x}=0$) are *y*=*a*_1_*t* and *y*=*a*_3_*t*, so the origin (*x*,*y*,*t*)=(0,0,0) is a two-fold. Via elementary calculations, we find that we need *a*_2_<*a*_1_, *a*_3_<*a*_4_ and *a*_1_≠*a*_3_ in order for the two-fold to be generic and for both folds to be invisible, as in (2.1). We also require *a*_1_<*a*_3_ in order to have *V*^−^,*V*^+^<0 and *V*^−^*V*^+^>1, as in (2.5). In summary, we require

5.4 $$a_{2}<a_{1}<a_{3}<a_{4}.$$

After some experimentation, we found the values

5.5 $$a_{1}=-0.2,\;a_{2}=-1,\;a_{3}=0.2\;\mathrm{and}\;a_{4}=1$$

to be particularly effective. With smaller values of the *a*_*i*_, larger negative values of *t*_1_ are required in order for the control to direct orbits into the two-fold; in that case the phase randomization is not achieved as quickly. With larger values of the *a*_*i*_, we observed distributions of the asymptotic phase that were substantially non-uniform; in that case the control does not ‘fully randomize’ the phase.

**Figure 9.**
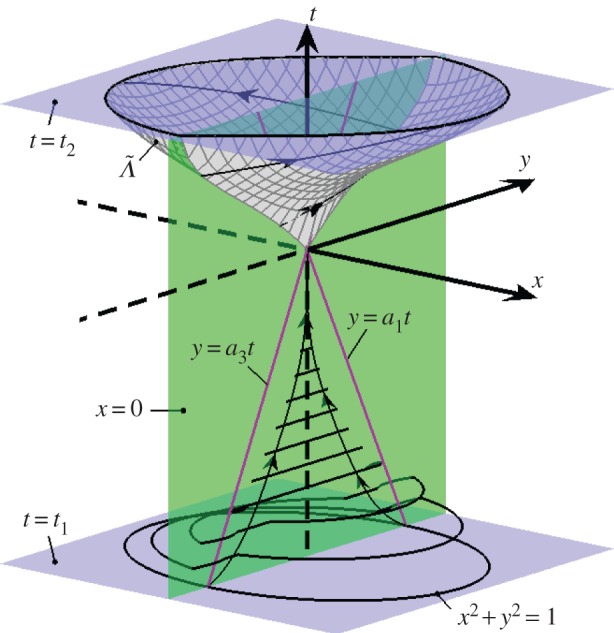
The dynamics of (5.2) for *t*_1_<*t*<*t*_2_ (when the control is active) in the absence of noise using (5.5), *t*_1_=−5, and *t*_2_=2.5. The evolution of *x*^2^+*y*^2^=1—the periodic orbit of (5.1)—is indicated for *t*_1_<*t*<0. As we evolve the set *x*^2^+*y*^2^=1, it contracts to the discontinuity surface at *t*≈−3.7, then to the two-fold at *t*=0. For 0<*t*<*t*_2_, viable Filippov solutions emanating from the two-fold evolve on $\tilde{\Lambda }$. (Online version in colour.)

We require *t*_1_<0 and |*t*_1_| to be sufficiently large that all points on the periodic orbit of (5.1) are directed into the two-fold from *t*=*t*_1_ to *t*=0. The size of the set of all points (*x*,*y*) that are directed into the two-fold increases with |*t*_1_|, and with (5.5) this set contains the periodic orbit for all *t*_1_≤−4.1, approximately. For this reason, we use *t*_1_=−5. The evolution of the periodic orbit from *t*=*t*_1_ to *t*=0 is shown in figure 9.

Finally, we would like the value of *t*_2_>0, at which time the control is turned off, to be small so that the phase randomization is achieved relatively quickly. However, with smaller values of *t*_2_ solutions lie nearer to (*x*,*y*)=(0,0) at *t*=*t*_2_. Since this point is an equilibrium of (5.1), smaller values of *t*_2_ cause solutions to dwell near (0,0) for longer periods of time, and so the phase randomization is not affected as quickly. Here we use *t*_2_=2.5 as it proves to be a suitably intermediate value. In figure 9, the unstable manifold $\tilde{\Lambda }$ is shown for *t*=0 to *t*=*t*_2_.

**Figure 10.**
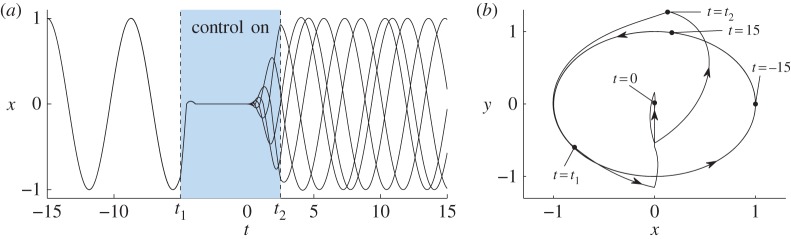
Panel (*a*) shows five sample solutions to (5.2) using (5.5), *t*_1_=−5, *t*_2_=2.5, *ε*=0.001 and the initial condition (*x*,*y*)=(1,0) at *t*=−15. The control (5.3) causes the solutions to pass close to a two-fold at *t*=0, effectively randomizing the phase. Panel (*b*) shows one of the solutions in phase space, with the location of the solution highlighted at key times. The sample solutions were computed using the Euler–Maruyama method with a step-size of Δ*t*=10^−5^. (Online version in colour.)

Figure 10 illustrates the phase randomization using (5.5), *t*_1_=−5 and *t*_2_=2.5. We see that the randomization appears to be highly effective. A quantitative comparison of our phase randomization with other methods remains for future work.

## 6. Discussion

In this paper, we have studied orbits that pass through an invisible two-fold (Teixeira singularity) then limit to a stable periodic orbit. We considered the presence of small noise in order to remove the ambiguity of evolution through the two-fold. We found that the phase of the limiting periodic motion is highly randomized and that the probability distribution of the phase depends crucially on the nature of the nonlinear dynamics experienced by orbits between the two-fold and the periodic orbit. By using polar coordinates to describe the motion of orbits as they initially depart from the two-fold, and a one-dimensional return map to describe the global dynamics approaching the periodic orbit, we constructed an approximation to the probability density function of the asymptotic phase that matches well to the results of numerical simulations (figure 1).

In §5, we showed how a simple discontinuous control law can generate a two-fold in a smooth system. We propose that in this fashion the two-fold can be used to desynchronize a collection of oscillators and that this may have the following advantages to desynchronization using an unstable equilibrium.

(i) With an unstable equilibrium, the control action must direct the state of each oscillator close to the equilibrium, and the effectiveness of the randomization correlates with the accuracy of this action. With a two-fold, however, the control does not need to be as precise, because it only needs to direct orbits to the basin of attraction of the two-fold.
(ii) With an unstable equilibrium, the effect of the randomization is due to inherent stochasticity in the system and any artificial randomness in the control law. With a two-fold, the randomization is inherent in the ambiguity of forward evolution through the two-fold.
(iii) Orbits dwell near unstable equilibria and so can take a relatively long time to return to regular periodic motion. With a two-fold there is no slowing of the dynamics, hence we refer to our desynchronization as ‘fast’ phase randomization. Indeed, in figure 10, we see that solutions return quickly to approximately regular periodic motion after the control is turned off. Whether this truly constitutes fast desynchronization requires a study of the full time for which the control action is required. This and a study of practical discontinuous control actions remain for future work.

Teixeira’s paper [10] inspired a legacy of intrigue around a specific case of the two-fold singularity, first studied more generally in [9]. Interest has only increased as its role as a determinacy-breaking singularity has become more clear [11,16]. This paper begins to reveal the practical side of these insights, including how two-folds may manifest in physical systems, and how they might be put to use as tools for control. Little is still known about where two-folds appear naturally in physical systems. Similarly, little is known about what applications they may have in control systems, but a role as a phase randomizer is suggested here. Perhaps the obvious next step is to design implementable control circuits to investigate the practical obstacles and opportunities that they present.

## Data accessibility

The figures in this paper are reproducible as we have provided all the necessary equations and parameter values in the text and figure captions.

## Appendix A. Proof of proposition 2.2

Proof. Since *ψ*_*a*_(*a*)∈*ζ*, the *y* and *r*-values of *ψ*_*a*_(*a*) are the same; see (2.23). The *y*-value of *ψ*_*a*_(*a*) is given by (2.17). By (2.29), the *r*-value of *ψ*_*a*_(*a*) is equal to *αa*. By matching these two expressions, we conclude that *α* is given by (2.27). At the beginning of §2b, we showed that, given *x*<0 and y∈R, if *τ*_*L*_>0 is given by (2.21) and *y*_0_=*y*−*V*^−^*τ*_*L*_, then evolving (2.1) forwards from (0,*y*_0_,*γy*_0_) for a time *τ*_*L*_ takes us to the point (*x*,*y*,*z*), where *x*=−*Ξ*(*y*,*z*)/*V*^−^ (2.15). Moreover, this defines a bijection from the region *x*<0, y∈R to the region *y*_0_>0, 0<*τ*_*L*_<−2*γy*_0_. By (2.24), evolving (*r*,*θ*)=(*y*_0_,0) for a time *τ*_*L*_ takes us to the point

A 1 $$(r,\theta )=\left(y_{0}+\alpha \tau_{L},\;\frac{\beta }{\alpha }\ln\left(\frac{\alpha \tau_{L}}{y_{0}}+1\right)\right).$$

This is a bijection from *y*_0_>0, 0<*τ*_*L*_<−2*γy*_0_ to *r*>0, 0<θ<(β/α)ln⁡(1−2αγ) (assuming *β*>0). By substituting (2.21) and *y*_0_=*y*−*V*^−^*τ*_*L*_ into (6.1) and simplifying, we arrive at (2.25) and (2.26) for *x*<0. Similarly, given *x*>0 and y∈R, let *τ*_*R*_<0 be given by (2.22) and *y*_0_=*y*−*τ*_*R*_. This is a bijection to *y*_0_>0, −2*y*_0_<*τ*_*R*_<0, and evolving (2.1) backwards from (0,*y*_0_,*γy*_0_) for a time *τ*_*R*_ takes us to (*x*,*y*,*z*), where *x*=*Ξ*(*y*,*z*)/*V*^+^ (2.15). In polar coordinates evolving (*r*,*θ*)=(*y*_0_,0) for a time *τ*_*R*_ takes us to

A 2 $$(r,\theta )=\left(y_{0}+\alpha \tau_{R},\;\frac{\beta }{\alpha }\ln\left(\frac{\alpha \tau_{R}}{y_{0}}+1\right)+2\pi \right),$$

which is a bijection to *r*>0, (β/α)ln⁡(1−2α)+2π<θ<2π. Substituting (2.22) and *y*_0_=*y*−*τ*_*R*_ into (6.2) leads to (2.25) and (2.26) for *x*>0. Finally, by matching the limiting left and right values for *θ* on the negative *y*-axis as given by (2.26), we obtain

A 3 $$\theta =\frac{\beta }{\alpha }\ln(1-2\alpha \gamma )=\frac{\beta }{\alpha }\ln(1-2\alpha )+2\pi .$$

By using the identity *μ*=(1−2*αγ*)/(1−2*α*) and solving (6.3) for *β*, we obtain the expression for *β* given in (2.27). This completes the proof, because the last calculation provides continuity of *θ*(*x*,*y*), and by construction *r* and *θ* satisfy the ordinary differential equations (2.24). ▪
